# Role of diagnostic imaging in psoriatic arthritis: how, when, and why

**DOI:** 10.1186/s13244-021-01035-0

**Published:** 2021-08-25

**Authors:** Ana María Crespo-Rodríguez, Jesús Sanz Sanz, Dalifer Freites, Zulema Rosales, Lydia Abasolo, Juan Arrazola

**Affiliations:** 1 Radiology Department, Hospital Clinico San Carlos, Madrid, Spain; 2 Faculty of Medicine, Complutense University of Madrid (UCM), Madrid, Spain; 3 Biomedical Imaging Research Group, Health Research Institute of the Hospital Clinico San Carlos, IdISSC, Madrid, Spain; 4 c/ Profesor Martín Lagos S/N, 28040 Madrid, Spain; 5 Reumathology Department, Hospital Puerta de Hierro Majadahonda, Majadahonda, Spain; 6 Reumathology Department, Hospital Clinico San Carlos, Madrid, Spain; 7 Research Group On Inflammation, Infection, Immunity and Allergy, Health Research Institute of the Hospital Clinico San Carlos (IDISSC), Madrid, Spain

**Keywords:** Psoriatic arthritis, Arthritis, Radiography, Ultrasound, Magnetic resonance imaging

## Abstract

Psoriasis is a common skin disease. Up to 30% of patients with psoriasis develop psoriatic arthritis (PsA) resulting, by far, the most prevalent coexisting condition. Heterogeneity of clinical and radiological presentation is a major challenge to diagnosis of PsA. Initial reports about PsA emphasized a benign course in most patients, but it is now recognized that psoriatic arthritis often leads to impaired function and a reduced quality of life. PsA is a progressive disease characterized by diverse clinical features, often resulting in diagnostic delay and treatment that are associated with poor clinical and structural outcomes. New effective treatments may halt PsA progression, and consequently, treatment goals have evolved from simple reduction of pain to achieving full remission or minimal disease activity. This emerging treat-to-target strategy paradigm emphasize a need for early diagnosis; sensitive imaging techniques may be of value in this process. While radiography and CT depict structural damage, US and MRI have emerged as helpful tools to evaluate magnitude and severity of active inflammatory lesions. This review aims to describe the role of imaging modalities in diagnosis, follow-up and prognosis of PsA.

## Key points

- Psoriatic arthritis is a heterogeneous disease with multiple musculoskeletal and dermatological manifestations.
- In up to 15% of patients, psoriatic arthritis precedes the skin disease or occur simultaneously.
- Identifying the early form of psoriatic arthritis (PsA) leads to better outcome.
- Patients with psoriasis can suffer other forms of arthritis beyond psoriatic arthritis, which must be excluded before treatment.
- Focus of research on efficient tools for diagnosis, monitoring and prognostication of PsA is due to introduction of new effective therapies and a treat-to-target-strategy.

## Background

**Fig. 1:**
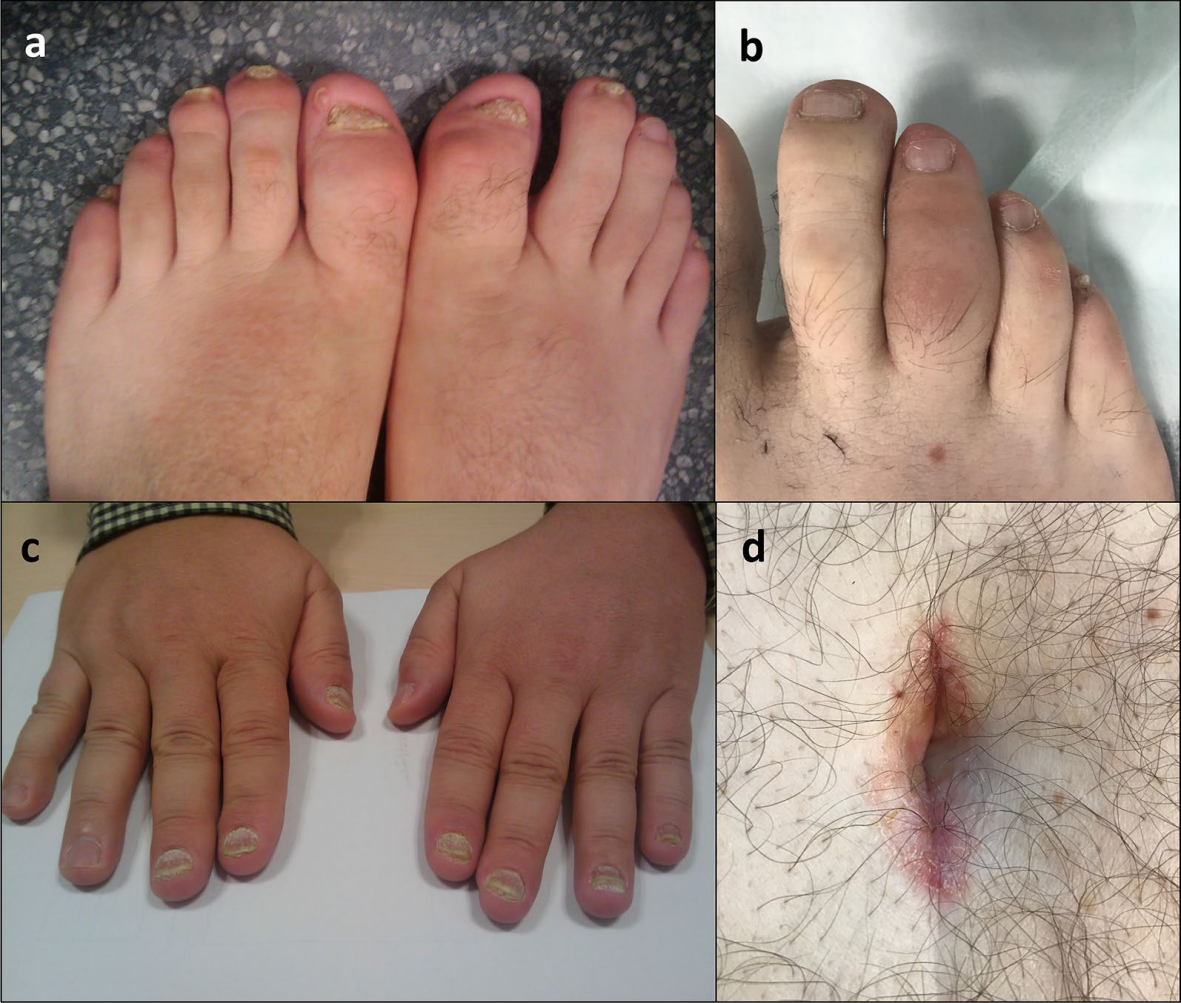
**a**, **c** Pitting and ridging of the nails is a common sign of psoriasis and also in psoriatic arthritis. **b** Dactylitis, uniform sausage-like swelling of the whole digit either finger or toe, is a hallmark clinical feature of PsA. **d** Umbilicus, as well as behind the ears and at the top of the natal cleft are hidden areas for skin psoriasis

Psoriasis is a common skin disorder characterized by the development of inflammatory plaques on the skin. The wide clinical spectrum of psoriasis (Fig. 1) includes chronic plaque, guttate, inverse, erythrodermic, pustular, and nail variants of the disease. Psoriatic arthritis is the most prevalent coexisting condition associated with psoriasis and it occurs in up to 30% of patients with psoriasis [1]. So, PsA is a common spondiloarthritis that can be found either in the peripheral or in the axial skeleton. The relationship between arthritis and the other domains of psoriasis is poorly explained by what is known about the etiopathogenic of psoriatic arthritis [2]. Beyond skin and joints involvement, a wide range of extra-articular manifestations and comorbilities may be present: cardiovascular disease, atherosclerosis, metabolic syndrome, nonalcoholic fatty liver disease, smoking, alcoholism, osteoporosis, and depression [1, 3].

No gender predilection has been observed in PsA [4]. PsA has an incidence of about 6 per 100,000 per year and a prevalence of approximately 1–2 per 1000 [4]. The wide variability of the reports about its incidence and the prevalence may be due to different definitions [5], as well as geography [6].

The annual incidence of PsA was reported to be 2–3% in a prospective study of patients with psoriasis [7]. Undiagnosed PsA occurs in up to one-third of patients with psoriasis who are followed by dermatologists [8, 9].

Usually, the skin manifestation of psoriasis precedes that of arthritis by 10 years on average. But an unexpected clinical scenario occurs in up to 15% of patients when arthritis and psoriasis occur simultaneously or psoriatic arthritis precedes the skin disease [1, 7].

Initial reports about PsA emphasized a benign course in most patients, but it is now recognized that psoriatic arthritis often leads to joint damage and a reduced quality of life [1, 3]. New effective treatments may halt this progression [10], and consequently, treatment goals have evolved from simple reduction of pain to achieving full remission or minimal disease activity [11]. This emerging treat-to-target strategy paradigm emphasize a need for early diagnosis [11]; sensitive imaging techniques may be of value in this process [12].

Our purpose is to review the role of imaging techniques in the diagnosis, follow-up and prognosis of psoriatic arthritis making emphasis on early acute inflammatory lesions on US and MRI.

## Diagnostic criteria

Without diagnostic criteria validated for psoriatic arthritis, the Classification Criteria for Psoriatic Arthritis (CASPAR criteria, Table 1), published in 2006, serves for the purpose of enrolling patients in clinical trials and provides guidance to clinicians [13]. The criteria were found to be easy and practical to apply with a sensitivity of 0.914 and specificity of 0.987, these findings have been confirmed with subsequent studies [14–18].

**Table 1:** The recently developed CASPAR (classification criteria for PsA) criteria consist of established inflammatory arthritis, defined by the presence of tender and swollen joints and prolonged morning or immobility-induced stiffness, with a total of at least 3 points from the features listed in this table

CASPAR criteria [13]		
Skin psoriasis	Present	2 points
	Previously present by history	1 point
	A family history of psoriasis	1 point
Nail lesions (Onycholysis, pitting)		1 point
Dactylitis (present or past, documented by a rheumatologist)		1 point
Negative rheumatoid factor (RF)		1 point
Juxtaarticular bone formation on radiographs (distinct from osteophytes)		1 point

Psoriatic arthritis is grouped with spondyloarthritis [19] by virtue of the genetic and clinical features shared with these disorders. The term spondyloarthritis (SpA) includes a group of diseases characterized by inflammation in the spine and in the peripheral joints, to which other specific clinical features are added such as uveitis, dactylitis, psoriasis, inflammatory bowel disease, and human leukocyte antigen (HLA) B27 [20]. This heterogenous group includes axial spondyloarthritis (ankylosing spondylitis) and peripheral spondyloarthritis, encompassing psoriatic arthritis (PsA), reactive arthritis and inflammatory bowel disease-associated arthritis. Recently, the Assessment of SpondyloArthritis International Society (ASAS) classification criteria has been developed [20]. Furthermore, lesions depicted on MRI were categorized into active inflammatory lesions and structural lesions. Active or acute inflammatory lesions encompass bone edema and osteitis, synovitis, enthesitis and capsulitis. Whereas structural lesions include subchondral sclerosis, erosions, periarticular fat bone marrow deposit, bone bridges and ankylosis [20].

The diagnosis of PsA can generally be made in a patient who has both psoriasis and an inflammatory arthritis in a pattern typical of PsA. Other forms of arthritis can occur in patients with psoriasis, such as rheumatoid arthritis, osteoarthritis, gout, reactive arthritis, and the arthritis of inflammatory bowel disease, and should be excluded as the cause of the patient’s syndrome, generally based upon the pattern of joint involvement, laboratory testing, imaging, and synovial fluid analysis. Certain clinical features may suggest PsA in the absence of psoriasis (psoriatic arthritis sine psoriasis), such as distal joint involvement, an asymmetric distribution, nail lesions, dactylitis, and the family history.

There is a lack of enough scientific evidence on the use of musculoskeletal imaging in the clinical management of PsA. In this situation, EULAR recommendations for the use of imaging in the diagnosis and management of spondyloarthritis in clinical practice could be implemented [21].

## Clinical manifestations of PsA and management of PsA

Patients with psoriatic arthritis (PsA) present with pain and stiffness in the affected joints.

Moll and Wright [22] precociously described five clinical scenarios:

- Distal arthritis, characterized by involvement of the DIP joints
- Asymmetric oligoarthritis, in which less than five small and/or large joints are affected in an asymmetric distribution.
- Symmetric polyarthritis.
- Arthritis mutilans
- Spondyloarthritis (SpA), including sacroiliitis.

The two most common between these five clinical subsets are asymmetric oligoarthritis and symmetric polyarthritis [22]. Because the clinical subsets described above can change over time in an individual patient, the clinical manifestations of PsA are most commonly described using the following six clinical domains [23]: Peripheral arthritis, axial disease, enthesitis, dactylitis, skin and nail disease. Beyond arthritis, periarticular disease is other common rheumatologic feature of PsA and includes tenosynovitis and soft tissue inflammation similar to that seen in other forms of SpA (or "seronegative arthritis"), such as enthesitis and dactylitis.

The day-to-day management of patients with PsA includes non-pharmacological and pharmacological interventions. The number of disease-modifying antirheumatic drugs (DMARDs) indicated for PsA has increased during the last year, including synthetic DMARDs such as methotrexate and sulfasalazine, but also other targeted biological agents aimed at different cytokines, such as TNF, interleukin-12/23 and IL-17A, as well as targeted synthetic DMARDs that inhibit phosphodiesterase-4 or Janus kinases. The goal of treating patients with PsA is to control of symptoms, prevention of structural damage, normalization of function and social participation in order to improve health-related quality of life [24–27].

## Imaging findings

**Fig. 2:**
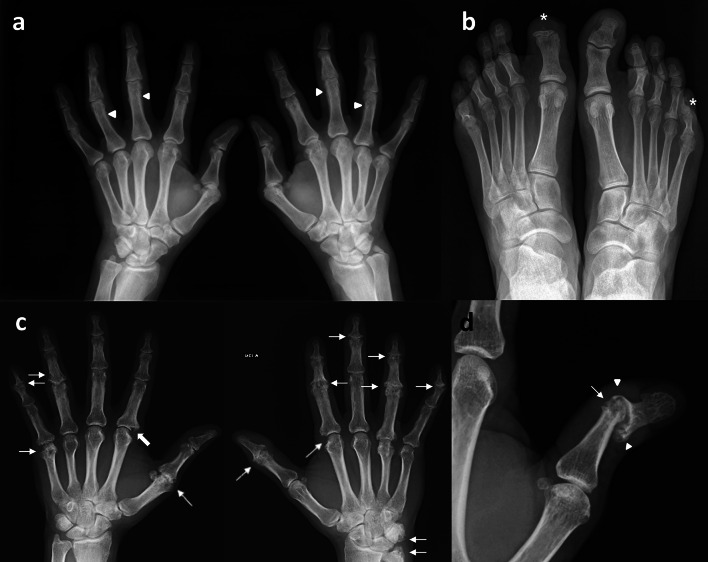
A characteristic patter of PsA is the coexistence of erosive changes and new bone formation as in this example where the same patient (**a**, **b**) presented with fluffy periotitis in the proximal phalanges in both hands (arrow head) and acro-osteolysis (*) in the distal phalanges of the first toe in the right foot and the fifth toe of the left foot. **c** Polyarticular and asymmetrical PsA on hands with interphalangeal joints involved showing articular space narrowing and erosions (thin arrows), one of them is a Ω shaped erosion (thick arrow). **d** Subluxation and pencil-in-cup appereance in interphalangeal joint of the thumb in this particular case is another example of typical radiological changes in peripheral PsA. Bony erosions narrowed the end of the proximal phalange as a “pencil” (thin arrows) which rested in “cup” formed by the expanded base of the adjacent phalange (arrow head)

Radiographic changes in the course of PsA exhibit a striking and characteristic pattern usually not seen in other forms of inflammatory arthritis, which is the coexistence of erosions and new bone formation [28] (Fig. 2a, b).

Radiography is a fast, feasible, reliable, and relatively inexpensive procedure. Thanks to the ability to provide a record of the cumulative joint damage [29], radiography is most widely used imaging exam in arthritis and in the particular case of PsA.

While radiography of peripheral joints requires a small dose of ionizing radiation, for evaluating the axial skeleton the required dose is greater. This concern in children and young adults contributes to favoring MRI [21].

Radiological damage was observed in at least a quarter of early PsA patients [30]. Althoutgh arthritis of longer duration than reported by the patient is a possibility, the presence of joint damage on radiography has been proved to be an independent predictor of radiographic progression [31] and so, of a very aggressive disease.

### Radiographic findings in Peripheral PsA

Joint involvement in peripheral PsA is highly variable, and often changes over time [32]. PsA can involve the small joints of hands and feet. Another frequent joint pattern is that of oligoarthritis affecting mainly larger weight bearing joints.

Erosive changes and new bone formation [28] may occur within the same joint or in different joints within the same digit. Unlike for Rheumatoid Arthritis (RA), bone density is normal and bilaterality and symmetry less frequent (Fig. 2c).

On the hands, two different patterns have been described for PsA:

- A “row pattern” when there is predilection for the distal interphalangeal joint and sparing of the metacarpophalangeal joint.
- A “ray pattern”, when all three joints in one digit are affected, and potentially also the wrist [23].

The “ray pattern” and asymmetric arthritis have been used to clinically distinguish PsA from RA [33]. However, at least half the patients had symmetrical (RA-like) polyarthritis [34].

A typical appearance, althougth not specific to PsA, is “Pencil in Cup Deformity” (Fig. 2d). A term of art referring to bony erosions in which narrowed end of metacarpal or phalanges (pencil) rests in the expanded end of the adjacent bone sharing the joint (cup). Up to 5% of cases develop arthritis mutilans [35].

Other typical radiological changes include lysis of the terminal phalanges (acro-osteolysis), fluffy periostitis, as well as new bone formation at the site of enthesitis; gross destruction of isolated joints; and the occurrence of both joint lysis and ankylosis in the same patient [24].

Enthesitis and dactylitis also are commonly seen, and can accompany any of the other manifestations [32] and in particular, a “ray pattern”.

### Radiographic findings in Axial PsA

**Fig. 3:**
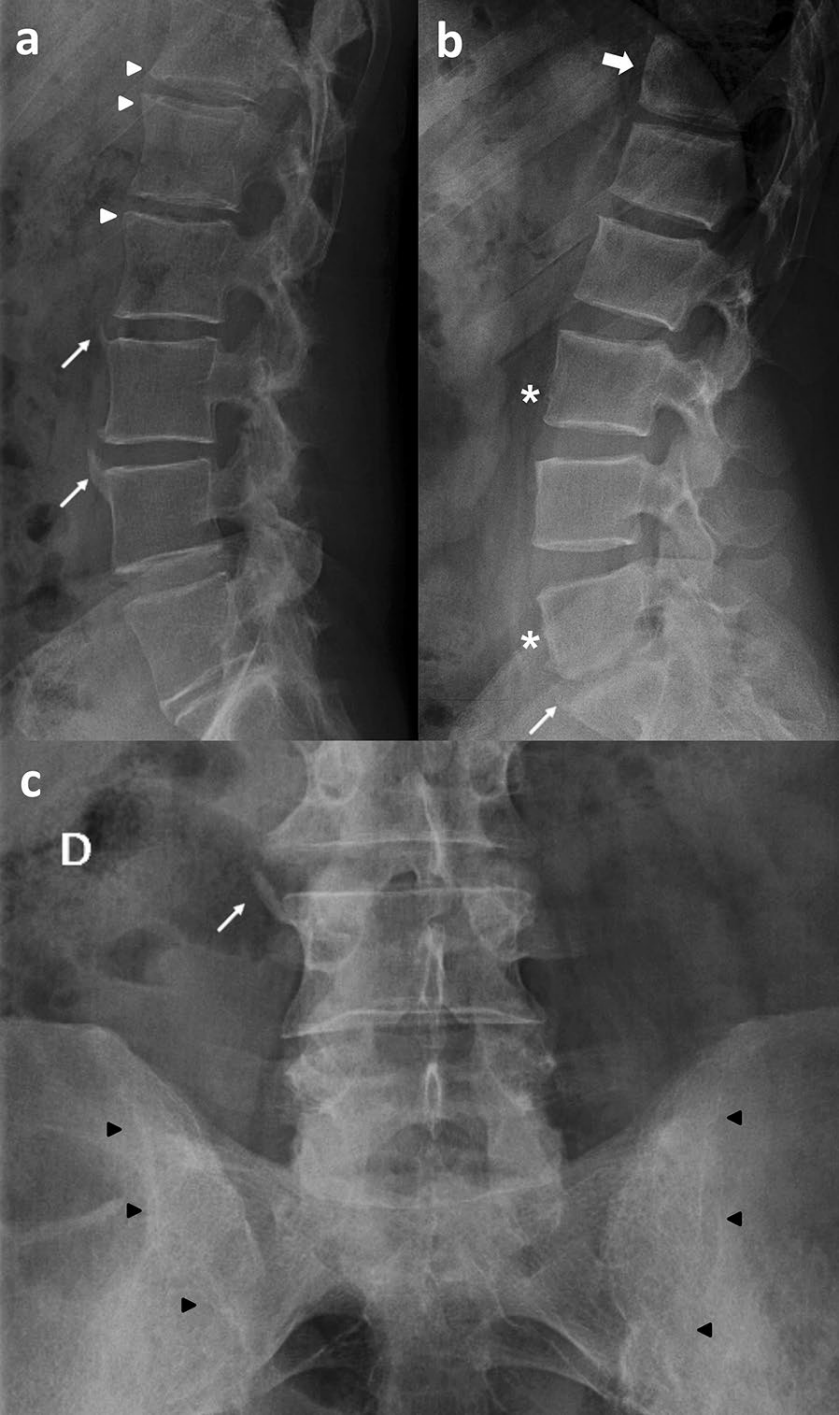
**a** Syndesmophytes at the anterosuperior endplate of L3 and L4 vertebrae (thin arrows) and Romanus lesions (erosions) at the anterior endplate of T12, L1 and L2 vertebrae (white arrow heads). **b** Square vertebrae (*) and barrel‐shaped vertebrae (thick arrow) show straightening or convex bulging of the ventral aspect of the vertebral body, mainly in the thoracolumbar junction and lumbar segments as a result of inflammation. **c** Right lateral syndesmophyte (thin arrow) at the superior endplate of L4 vertebra and sacroiliac ankyloses (black arrow heads)

In the spine PsA can have a slightly different pattern of radiographic features than axial Spondiloarthritis (SpA). Syndesmophytes are often bulkier than those seen in SpA, may be paramarginal, asymmetric and may skip vertebral levels. Better seen on anteroposterior view than on lateral view and more often thoracolumbar than cervical (Fig. 3). Radiography of the spine can be used for monitoring cumulative changes, particularly new bone formation [21].

In the sacro-iliac joints (SIJ), axial radiographs in patients with PsA may reveal changes identical to those seen in ankylosing spondylitis with symmetric sacroiliitis. However, asymmetric sacroiliitis, is highly suggestive of PsA.

It is recommended radiography of the SIJ as first line modality for diagnosis of sacroiliitis as part of axial SpA, followed by MRI if radiography is negative/inconclusive [21]. However, MRI of the SIJ is the preferred initial imaging method in patients with short disease duration and young patients [21].

Computed tomography (CT) is excellent for bone evaluation depicting bone erosion, sclerosis, and joint space alterations (from narrowing to ankylosis). A PsA pattern that differs from that observed in common osteoarthritis is new bone formation at entheses in psoriatic patients either with or without arthritis [36]. A recent publication revealed Ω-shaped erosions in the hands of PsA patients as opposed to U-shaped erosions noted in RA patients [37]. In the spine, the classical “shiny corner” sign, known as Romanus lesions, is a nonspecific spinal finding, representing reactive sclerosis secondary to inflammatory erosions at the vertebral bodies corners on lateral view radiograph [38]. Eventually, the vertebral bodies become squared, again another nor specific sign.

**Fig. 4:**
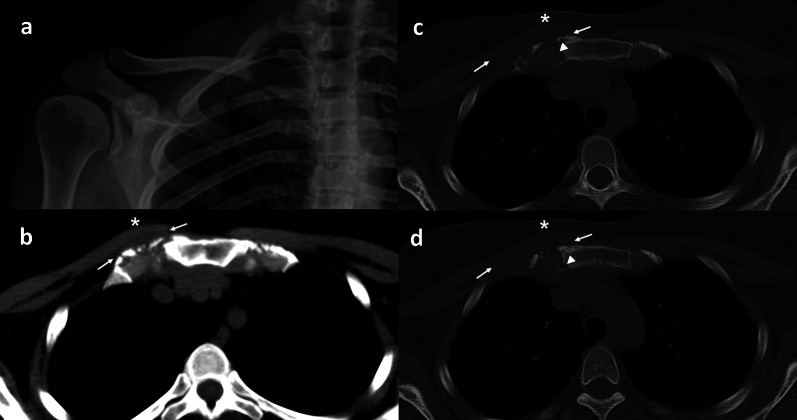
Right first sternochondral psoriatic arthritis in a 36-year-old woman. CT adds valuable information in anatomical complex areas like in this patient with normal X-ray examination of the right shoulder girdle including the right sternoclavicular joint (**a**). Axial CT images, either on soft tissue windowing (**a**) or bone algorithm (**c**, **d**) show asymmetry (*) and the loss of definition of the surrounding fat planes (thin arrows) between the first sternochondral joint and the muscles can also be appreciated. Small erosions in the sternal side are also visible (arrow heads)

CT is very useful in depiction of complex and difficult anatomical areas such as shoulder girdle (Fig. 4), spine, pelvis and in particular the SIJ.

CT could be considered the standard reference for assessing structural damage in spondiloarthritis (SpA). However, failure to detect active inflammatory lesions in addition to the ionizing radiation provided determine that the role of the CT in clinical practice is restringed to patients with a major contraindication for MRI [29].

Ultrasound (US) examinations are usually performed on B-mode and combination with power Doppler is highly recommended although it is dependent on the equipment. No ionizing radiation is involved.

US is a useful technique for the evaluation of inflammatory changes in soft tissues such as synovium, tendons, bursas and entheses, perfusion in joints and structural changes in the bone surface (erosions). The major limitation of US is full bone evaluation as it cannot penetrate bone and it is not sensitive for axial disease manifestations.

**Fig. 5:**
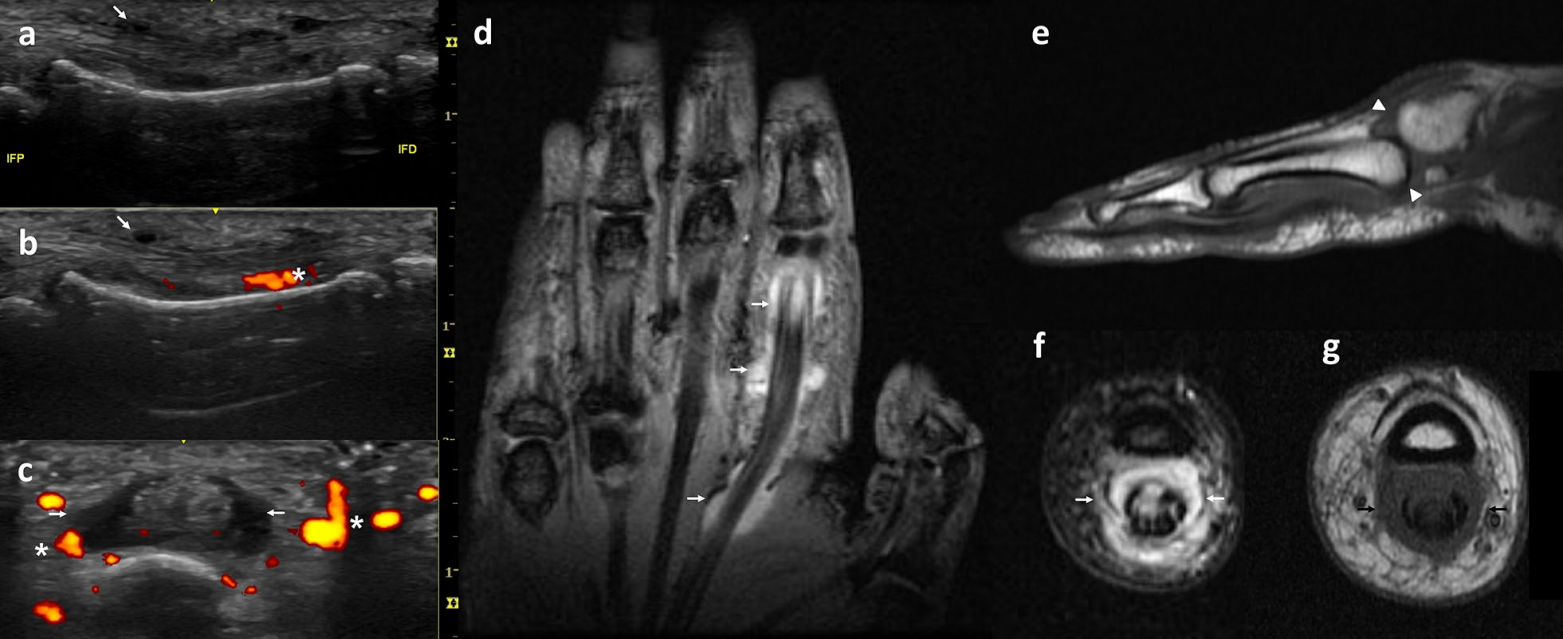
A 48-year-old woman presenting with dactylitis of the second finger of the hand. US examination (**a**–**c**) shows a hypoechoic swelling surrounding the flexor digitorum tendons (thin arrow) with Power Doppler signal showing hypervascularity (*) related to tenosynovitis of flexor tendon of the second finger on longitudinal (**a**, **b**) and axial (**c**) views. MR examination adds a general view of the hand in this coronal STIR wi (**d**) that shows fluid surrounding the second flexor digitorum tendons (thin arrows). Sagittal T1 wi (**e**) shows subluxation of the second carpo-metacarpal joint (arrow heads). Axial views weigthed on STIR (**f**) and T1 (**g**) show extensive tenosynovitis (thin arrows) of the flexor tendons

**Fig. 6:**
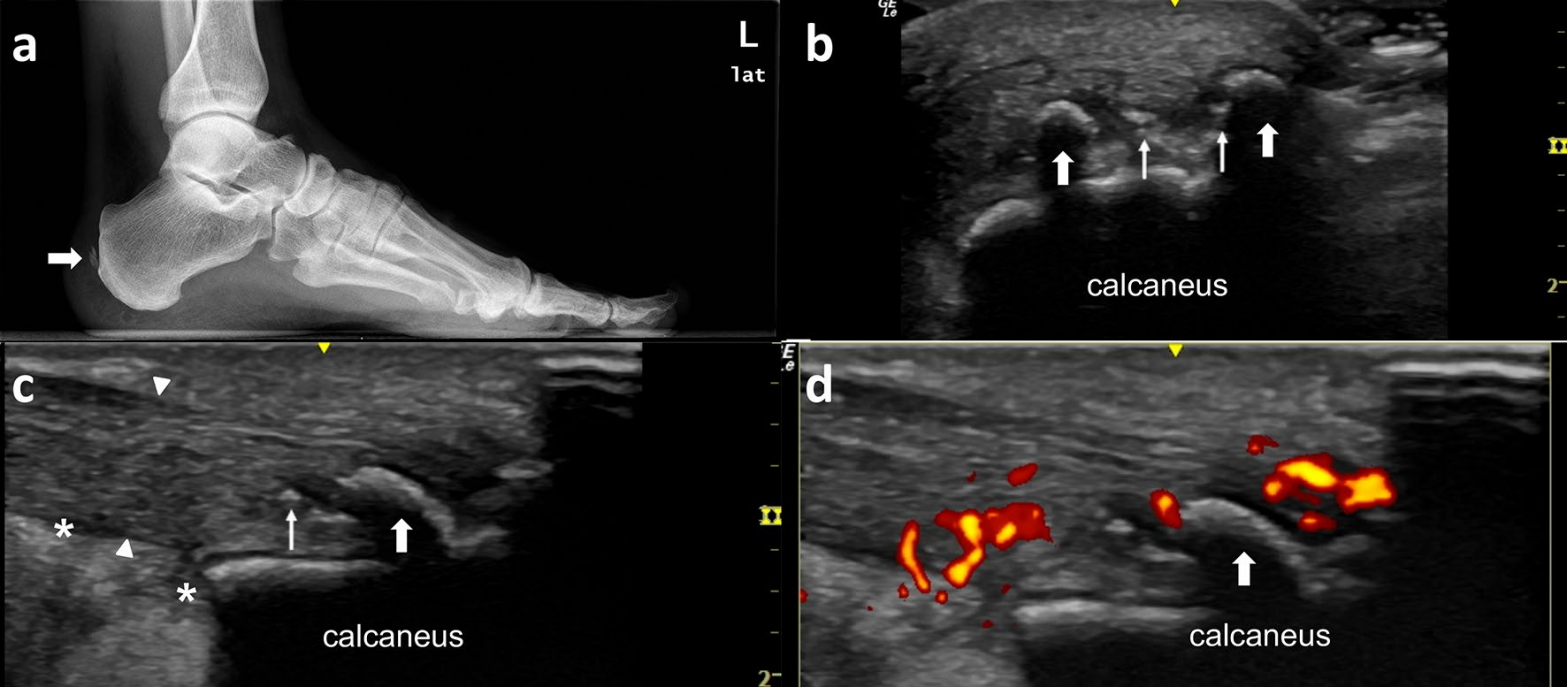
The Achilles tendon is among the most frequent sites of enthesopathic involvement in PsA. Forty-two-year-old male presenting with heel pain and difficulty walking. **a** Lateral X-Ray film shows a gross calcifications (thick arrow) at the distal Achilles tendon. These calficiations do not contact with the calcaneous bone and margins are ill-defined. On US examinations (**b**) these gross calcifications are identified as hyperechogenic surface with a posterior shadow (thick arrows) while other puntiforms hyperechogenic foci (thin arrow) do not have shadow and seems to be incipient calcification. **c** The Achilles tendon insertion is diffuse thickened (arrow head) and there is some fluid (*) at Kagger’s fat pad. **d** Color Doppler examination shows hypervascularity at the thickened Achilles tendon insertion at the calcaneous

In peripheral PsA, US is more sensitive than X-ray, scintigraphy or MRI and even in combination with clinical examination in PsA patients [39–41] for the detection of joint involvement, both intraarticular (synovitis and erosions) and extra-articular, including bursitis, tenosynovitis (Fig. 5), and enthesitis (Fig. 6).

Synovitis of peripheral joint in PsA is nonspecific and its solely diagnostic value in detecting joint inflammation [21].

Tenosynovitis is easily identified on US examination [42, 43] as a hypoechoic swelling surrounding the extensor digitorum tendon with or without power Dopppler signal (Fig. 5). Flexor tenosynovitis is recognized as the major contributor to clinical dactylitis [42, 43]. Recently, peritendon extensor tendon inflammation has emerged as another cause of metacarpophalangeal joint swelling, demonstrating to be a specific feature of PsA, of value in the differential diagnosis with other inflammatory diseases [45, 46].

Enthesitis is considered a pathophysiologically important feature of SpA and in the particular case of PsA [41]. There are two types of entheses: fibrous and fibrocartilaginous [47]. Fibrous enthesis are the insertions of the annulus of the disk in membranous bone of the vertebral endplates by Sharpey's fibers. Fibrocartilaginous enthesis are the connection of hyaline cartilage to endochondral or subchondral bone. Enthesitis is characterized by inflammation at sites of tendon, ligament, and joint capsule fiber insertion into bone. In 2014, a consensus based definitions for US elementary lesions for enthesitis independent of spondyloarthritis (SpA) type was developed [48]. These elementary lesions of enthesitis included presence of enthesophytes, calcifications (Fig. 6), and erosions at the insertion site (chronic changes) and increased thickness, hypoechogenicity, and Doppler activity in the enthesis (inflammatory changes) [48].

Recent registry and clinical trial patient sets have found enthesitis occurs in approximately 30–50% of PsA patients [48]. Enthesitis can involve almost any part of the body, although classically it is been depicted involving the Achilles tendon and plantar fascia insertion sites. Some locations are more accessible to US examination (periknee, pelvis, shoulder, and elbow) than others. Several enthesitis scoring measures have been developed, some originally developed in patients with ankylosing spondylitis (AS), as the Spondyloarthritis Research Consortium of Canada (SPARCC) enthesitis index [49]. While the Leeds enthesitis index (LEI) is specifically for PsA [50].

Entheseal involvement in patients with psoriasis, but without clinical PsA, suggests that enthesitis may be a predictor of development of PsA [51, 52]. However, the prognostic value of US in PsA needs further investigation. Role of US examination in PsA includes diagnosing peripheral involvement and monitoring disease activity.

Magnetic resonance imaging (MRI) can visualize all peripheral and axial joints and entheses involved in PsA, in order to assess inflammation and structural damage in detail.

A phased array body coil is used when imaging the spine and /or SIJ. The basic image acquisition protocol used for the spine routine imaging in MR are sagittal T1-weighted images (wi), sagittal T2 wi, axial T1 wi and axial T2 wi. For diagnostic of PsA, a spine protocol designed for axSpA is required. Fluid sensitive sequences such as fat-suppressed T2 wi and STIR, as well as post-gadolinium fat-suppressed T1-weighted sequence should be implemented [53]. There are few reports about the role of other sequences, such as diffusion wi [54] and dynamic contranst enhanced (DCE) imaging. Furthermore, the role of MRI sequence based on chemical, specifically designed to achieve uniform fat suppression, needs to be defined.

Sacroiliac MRI benefits from a small field-of-view of the sacroiliac joint imaging over large large-field-of-view of the pelvis. Sacroiliac routine imaging in MR is usually orientated to inflammation depiction. Imaging adquisition should take as reference the posterior vertebral wall of S2. Routine protocol should perform oblique coronal (T1 wi and T2 wi fat-sat) and oblique axial sequences (T1 wi, T2 wi fat-sat and T1 wi fat-sat postcontrast) images [55]. A screening protocol for sacroiliitis could be limited to oblique coronal “fat-sensitive” T1 wi and “fluid-sensitive” T2 wi fat-saturated sequences [55]. Post-contrast imaging when ruling out inflammatory sacroiliitis would not be mandatory [56].

MRI is highly sensitive in detecting articular, periarticular, and soft tissue inflammation [57–60]. However, signs of inflammation such as synovitis, tenosynovitis and bone marrow edema, are not specific for PsA. Clinical usefulness of MRI in PsA relies on the assessment of inflammation and structural damage of PsA. Distinction between active lesions and chronic lesions is essential for an accurate management of patient’s disease. Future imaging studies need to define the threshold for clinically significant inflammatory findings.

**Fig. 7:**
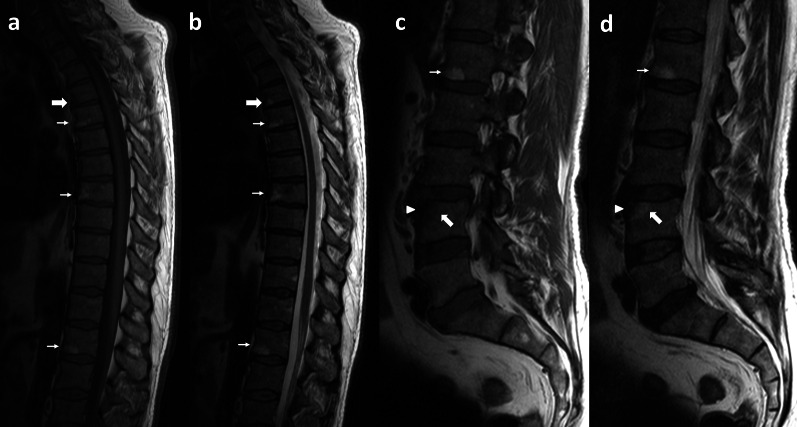
Sixty-year-old male with axial and peripheral PsA. Psoriatic spondiloarthropathy in this particular case presents with skip Romanus lesions at different stages. All of them shows bone erosion at the vertebral body corners. Signal intensity of adjacent bone reveals chronology of the lesion: Acute Romanus lesions at the anteroinferior endplate of T4 vertebra shows low signal intensity on T1 wi (**a**) and high signal intensity on T2 wi (**b**) due to bony edema. Subacute Romanus lesions at the anteroinferior endplate of T5, T8 and L1 vertebra shows high signal intensity on T1 wi (**a**, **c**) and high signal intensity on T2 wi (**b**, **d**) in relation to fatty replacement. Romanus lesion at the anterosuperior endplate of L4 vertebra shows chronic features with erosion and sclerosis (arrow head) and peripherally inflammatory changes with bone edema (thick arrow)

Detection on MRI of inflammation of entheses in joints felt to be clinically not inflamed, suggest that enthesitis may be the primary lesion in PsA, although this interpretation is controversial [61]. In the spine there are fibrous entheses, at the insertion of Sharpey's fibers to the vertebral end plate (membranous bone) [47, 62]. Imaging of early changes, as subentheseal bone marrow, is only achieved by MRI [47]. One of the earliest signs in the spine of inflammatory spondyloarthritis is Romanus lesion. It is characterized by inflammatory changes at the insertion of the annulus of the disk to the vertebral endplate [47, 62] like a fibrous enthesitis. This is more often seen anteriorly but can also be posterior or a combination of both. The changes seen are progressive (Fig. 7), beginning with acute inflammatory change and erosion at the vertebral body corners (low T1 and high T2 signal), replaced by fatty signal (high T1 and T2 signal) and eventually sclerosis (low T1 and T2). Bennett and colleagues described the fatty signal Romanus lesions in the spine, and they suggest may be the post-inflammatory phase between osteitis on MRI and sclerotic bone formation on radiographs [63]. This healing response to these inflammatory erosions appears radiographically as reactive sclerosis, which is known as the “shiny corner” sign. Just as MR imaging is better than conventional radiography in its depiction of Romanus lesions, MR imaging also provides a superior view of spondylodiscitis, since the edematous changes in early disease are not radiographically visualized.

The relationship between inflammation and new bone formation in PsA remains unclear. Recent MRI studies pointed out active corner inflammatory lesions as precursors of syndesmophytes [64] given their development at the exact location of resolved inflammatory lesions. Spinal involvement in PsA tends to be “spotty” and asymmetric with the addition of asymmetric chunky syndesmophytes and erosive discovertebral (Andersson) lesions [65, 66]. Andersson lesions are depicted as central portion disk-related signal intensity abnormalities of one or both vertebral halves of a discovertebral unit hyperintense, on STIR wi and hypointense on T1 wi, usually shaped as a hemisphere. On long TR sequences, hyperintense lines may be seen at the interface between the annulus fibrosus and nucleus pulposus in early disease.

MRI is more sensitive than routine radiography in detecting articular, periarticular, and soft tissue inflammation. Findings on MRI in the axial sacroiliac joints include joint effusion, bone marrow edema in the iliac and sacral bones, erosions, chronic changes in periarticular fat accumulation, sclerosis, and new bone formation [65]. Sacroillitis in PSA can be symmetric or asymmetric and sacroiliac ankylosis is infrequent. Asymmetric sacroiliitis is characteristic of PsA and may be asymptomatic. While MRI findings suggestive of moderate to severe sacroiliitis are found in more than one-third of patients, these imaging findings are poor predictors of clinical symptoms; MRI evidence of sacroiliitis does correlate with decreased spinal mobility and with longer duration of disease [67].

MRI can also provide detailed soft tissue and bone images of the chest wall and sternoclavicular region, an area frequently involved in PsA [68].

PsA is associated with a decrease in bone mass, as indicated by bone mineral density testing; this may lead to osteoporosis and to an increased risk of fractures [69].

## Differential diagnosis

The differential diagnosis should be made based on the radiological findings as X-ray examinations of joints are performed routinely when there is suspicion of PsA [70].

Lesions reflecting structural damage such as subchondral sclerosis, erosions, bone bridges and ankylosis of the joints are still graded primarily by X-ray examination but are also visible on MRI [20, 71, 72].

In the presence of joint space narrowing, first step is differentiating inflammatory arthritis from a degenerative arthritis [73]. Asymmetric distribution, presence of osteophytes and sclerosis are typically present in osteoarthritis [74].

Distribution of the findings, the so-called “target area approach to arthritis” by Resnick, allow distinction between the different types of arthritis [73].

- If inflammation involves a single joint, one must carefully exclude septic arthritis. In infectious spondylodiskitis or sacroiliitis surrounding soft tissue usually is involved and so, identification of an abscess in the soft tissue adjacent (paraspinal or epidural) is helpful for differential diagnosis [75].
- If inflammatory arthritis is diffuse and involves the proximal joints of the hands and feet without bone proliferation, rheumatoid arthritis is most likely [73].
- Distal involvement in hands and feet with bone proliferation suggests the presence of one of the seronegative spondyloarthropathies [73]. Differentiation among these disorders (psoriatic arthritis, Reiter syndrome and ankylosing spondylitis) largely relies on the distribution of radiographic abnormalities and clinical information. Reiter syndrome is a reactive sterile inflammatory arthritis that follows an infection at a different site, commonly enteric or urogenital [76]. An association with urethritis and conjunctivitis, as well as seropositivity for the HLA-B27 antigen, has been described [77]. Ankylosing spondylitis is an idiopathic inflammatory arthritis, with 96% of patients are HLA-B27 positive [77], commonly involves the axial skeleton, although peripheral joints may also be affected. Sacroiliac joint disease is bilateral and symmetric and it usually precedes spinal involvement. Spine involvement is characterized by osteitis, syndesmophyte formation, facet inflammation, and eventual facet joint and vertebral body fusion.

The differential diagnosis for bone production at the vertebral margins includes diffuse idiopathic skeletal hyperostosis (DISH), characterized by exuberant, flowing ossification along the anterolateral aspects of at least four contiguous vertebral bodies [78]. The absence of facet joint, costovertebral joint, and sacroiliac joint ankylosis, erosions, and sclerosis is very helpful to differentiate DISH from spondyloarthritis [78].

## Novel concepts and challenges in psoriatic arthritis

Formerly considered a milder form of arthritis, PsA is nowadays recognized as a progressive inflammatory musculoskeletal disease which if untreated leads to impaired function and long-term adverse outcomes [79]. More effective treatments available represent an opportunity for preventing PsA development from an early stage [80].

The pharmacological interventions of PsA includes different DMARDs and there is a growing body of evidence to support the implementation of a treat-to-target (T2T) strategy, using a pre-defined target in PsA management, with significant benefits in disease outcome, physical function, structural damage and quality of life. However, given the highly heterogeneous nature of PsA, there is poor agreement as to which target of response should be utilized, and incorporating T2T into clinical practice remain a challenge [81].

The presence of radiologic changes early in the course of PsA suggests either very aggressive disease or arthritis of longer duration than that reported by the patient [30, 82]. Kane et al. has reported, that joint erosions were present in 27% of patients at 10 months and in 47% of patients within 2 years of disease onset [83]. Peripheral joint disease is progressive in the majority of patients with the highest rate of progression in the first year of disease [80]. A prompt diagnosis of PsA is the first step toward optimal patient management given the propensity for the early occurrence of destructive disease. Prevalence of undiagnosed PsA remain high [79] despite the awareness of primary care physician and dermatologist. Delay in diagnosis in turn delays introduction of appropriate disease-modifying treatment and may contribute to poor patient outcome.

Early psoriatic arthritis is a heterogeneous condition that can make diagnosis difficult. Oligoarthritis of peripheral joints in a patient with plaque psoriasis and nail disease remains a common presenting scenario. However, the early stages of other forms of psoriatic arthritis may be difficult to distinguish, not only from other inflammatory joint disease such as rheumatoid arthritis, but even more so from osteoarthritis, fibromyalgia, and mechanical back pain.

In a study of early psoriatic arthritis, the presence of enthesitis, inflammatory low back pain, and dactylitis were helpful diagnostic features [84]. Heterogeneity of clinical presentation in early psoriatic arthritis is a major challenge to development of treatment algorithms [85].

In this sense, on the PsA research agenda is to further investigate the spatial and temporal relation between different imaging findings, providing further insight into the disease process, which may inform future clinical management of PsA and to investigate the importance of subclinical (detected only on imaging) peripheral inflammation (including bone marrow oedema, synovitis, tenosynovitis and/or enthesitis), and if possible, identifying thresholds to guide intervention. Consequently, to investigate incorporating such thresholds into T2T strategies [21].

## Conclusion

The importance of evaluating the magnitude and severity of active inflammatory lesions either on US or MRI has already been highlighted due to its value in the patient prognosis and in the assessment of the response to high cost of biological therapies. However, the precise nature of accurate and cost-effective screening strategies remains to be determined.

## Data Availability

Data sharing is not applicable to this article as no datasets were generated or analyzed during the current study.

## Abbreviations

CASPAR: Classification criteria for psoriatic arthritis
CT: Computed tomography
DIP: Distal inter-phalangeal
MRI: Magnetic resonance imaging
PsA: Psoriatic arthritis
RA: Rheumatoid arthritis
SIJ: Sacroiliac joints
SpA: Spondyloarthritis
US: Ultrasound
wi: Weighted images

## References

[1] Ritchlin CT, Colbert RA, Gladman DD (2017). Psoriatic arthritis. N Engl J Med.

[2] Veale DJ, Fearon U (2018). The pathogenesis of psoriatic arthritis. Lancet.

[3] Nijsten T, Wakkee M (2009). Complexity of the association between psoriasis and comorbidities. J Invest Dermatol.

[4] Gladman DD (2002). Current concepts in psoriatic arthritis. Curr Opin Rheumatol.

[5] Alamanos Y, Voulgari PV, Drosos AA (2008). Incidence and prevalence of psoriatic arthritis: a systematic review. J Rheumatol.

[6] Nossent JC, Gran JT (2009). Epidemiological and clinical characteristics of psoriatic arthritis in northern Norway. Scand J Rheumatol.

[7] Eder L, Haddad A, Rosen C (2016). The incidence and risk factors for psoriatic arthritis in patients with psoriasis: a prospective cohort study. Arthritis Rheumatol.

[8] Villani AP, Rouzaud M, Sevrain M (2015). Prevalence of undiagnosed psoriatic arthritis among psoriasis patients: systematic review and meta-analysis. J Am Acad Dermatol.

[9] Haroon M, Kirby B, Fitzgerald O (2013). High prevalence of psoriatic arthritis in patients with severe psoriasis with suboptimal performance of screening questionnaires. Ann Rheum Dis.

[10] Goulabchand R, Mouterde G, Barnetche T, Lukas C, Morel J, Combe B (2014). Effect of tumour necrosis factor blockers on radiographic progression of psoriatic arthritis: a systematic review and meta-analysis of randomised controlled trials. Ann Rheum Dis.

[11] Gossec L, Smolen JS, Ramiro S (2016). European League Against Rheumatism (EULAR) recommendations for the management of psoriatic arthritis with pharmacological therapies: 2015 update. Ann Rheum Dis.

[12] Mathew AJ, Coates LC, Danda D, Conaghan PG (2017). Psoriatic arthritis: lessons from imaging studies and implications for therapy. Expert Rev Clin Immunol.

[13] Taylor W, Gladman D, Helliwell P et al (2006) Classification criteria for psoriatic arthritis: development of new criteria from a large international study. Arthritis Rheum 54:2665–2673 10.1002/art.21972 16871531

[14] Chandran V, Schentag CT, Gladman DD (2007). Sensitivity of the classification of psoriatic arthritis criteria in early psoriatic arthritis. Arthritis Rheum.

[15] Chandran V, Schentag CT, Gladman DD (2008). Sensitivity and specificity of the CASPAR criteria for psoriatic arthritis in a family medicine clinic setting. J Rheumatol.

[16] Leung YY, Tam LS, Ho KW (2010). Evaluation of the CASPAR criteria for psoriatic arthritis in the Chinese population. Rheumatology.

[17] Coates LC, Emery P, Green P, Ibrahim M, Maklver GH, Helliwell P (2011) Investigating the use of the CASPAR criteria in early psoriatic arthritis [abstract]. EULAR 2011 Congress London. Rheumatology; iii144

[18] Tillett W, Costa L, Jadon D (2012). The ClASsification for Psoriatic ARthritis (CASPAR) criteria: a retrospective feasibility, sensitivity, and specificity study. J Rheumatol.

[19] Garg N, van den Bosch F, Deodhar A (2014). The concept of spondyloarthritis: where are we now?. Best Pract Res Clin Rheumatol.

[20] Rudwaleit M, Jurik AG, Hermann KG (2009). Defining active sacroiliitis on magnetic resonance imaging (MRI) for classification of axial spondyloarthritis: a consensual approach by the ASAS/OMERACT MRI group. Ann Rheum Dis.

[21] Mandl P, Navarro-Compan V, Terslev L (2015). EULAR recommendations for the use of imaging in the diagnosis and management of spondyloarthritis in clinical practice. Ann Rheum Dis.

[22] Wright V, Moll JM (1971). Psoriatic arthritis. Bull Rheum Dis.

[23] Ritchlin CT, Kavanaugh A, Gladman DD (2009). Group for Research and Assessment of Psoriasis and Psoriatic Arthritis (GRAPPA). Treatment recommendations for psoriatic arthritis. Ann Rheum Dis.

[24] Gossec L, Baraliakos X, Kerschbaumer A (2020). EULAR recommendations for the management of psoriatic arthritis with pharmacological therapies: 2019 update. Ann Rheum Dis.

[25] Kavanaugh A, Husni ME, Harrison DD (2017). Safety and efficacy of intravenous golimumab in patients with active psoriatic arthritis: results through week twenty-four of the GO-VIBRANT study. Arthr Rheumatol.

[26] Mease PJ, van der Heijde D, Ritchlin CT (2017). Ixekizumab, an interleukin-17A specific monoclonal antibody, for the treatment of biologic-naive patients with active psoriatic arthritis: results from the 24-week randomised, double-blind, placebo-controlled and active (adalimumab)-controlled period of the phase III trial SPIRIT-P1. Ann Rheum Dis.

[27] Mease P, Hall S, FitzGerald O (2017). Tofacitinib or adalimumab versus placebo for psoriatic arthritis. N Engl J Med.

[28] Siannis F, Farewell VT, Cook RJ, Schentag CT, Gladman DD (2006). Clinical and radiological damage in psoriatic arthritis. Ann Rheum Dis.

[29] van der Heijde D, Østergaard M, Bijlsma JWJ (2009). Assessment of disease activity and damage in inflammatory arthritis. The EULAR compendium on rheumatic diseases.

[30] Kane D, Stafford L, Bresnihan B, FitzGerald O (2003). A prospective, clinical and radiological study of early psoriatic arthritis: an early synovitis clinic experience. Rheumatology (Oxford).

[31] van der Heijde D, Landewé R (2005). Selection of a method for scoring radiographs for ankylosing spondylitis clinical trials, by the Assessment in Ankylosing Spondylitis Working Group and OMERACT. J Rheumatol.

[32] Gladman DD, Antoni C, Mease P, Clegg DO, Nash P (2005). Psoriatic arthritis: epidemiology, clinical features, course, and outcome. Ann Rheum Dis.

[33] Martel W, Stuck KJ, Dworin AM, Hylland RG (1980). Erosive osteoarthritis and psoriatic arthritis: a radiologic comparison in the hand, wrist, and foot. AJR Am J Roentgenol.

[34] Chandran V, Stecher L, Farewell V, Gladman DD (2018). Patterns of peripheral joint involvement in psoriatic arthritis-Symmetric, ray and/or row?. Semin Arthr Rheum.

[35] Haddad A, Johnson SR, Somaily M et al (2015) Psoriatic arthritis mutilans: clinical and radiographic criteria. a systematic review. J Rheumatol 42:1432–1438 10.3899/jrheum.141545 26077409

[36] Simon D, Faustini F, Kleyer A (2016). Analysis of periarticular bone changes in patients with cutaneous psoriasis without associated psoriatic arthritis. Ann Rheum Dis.

[37] Finzel S, Englbrecht M, Engelke K, Stach C, Schett G (2011). A comparative study of periarticular bone lesions in rheumatoid arthritis and psoriatic arthritis. Ann Rheum Dis.

[38] Romanus R, Yden S (2003). Destructive and ossifying spondylitic changes in rheumatoid ankylosing spondylitis (pelvo-spondylitis ossificans). Acta Orthop Scand.

[39] Wiell C, Szkudlarek M, Hasselquist M (2007). Ultrasonography, magnetic resonance imaging, radiography, and clinical assessment of inflammatory and destructive changes in fingers and toes of patients with psoriatic arthritis. Arthr Res Ther.

[40] Galluzzo E, Lischi DM, Taglione E (2000). Sonographic analysis of the ankle in patients with psoriatic arthritis. Scand J Rheumatol.

[41] Milosavljevic J, Lindqvist U, Elvin A (2005). Ultrasound and power Doppler evaluation of the hand and wrist in patients with psoriatic arthritis. Acta Radiol.

[42] Kane D, Greaney T, Bresnihan B, Gibney R, FitzGerald O (1999). Ultrasonography in the diagnosis and management of psoriatic dactylitis. J Rheumatol.

[43] Gutierrez M, Filippucci E, De Angelis R, Filosa G, Kane D, Grassi W (2010). A sonographic spectrum of psoriatic arthritis: “the five targets”. Clin Rheumatol.

[44] Gutierrez M, Filippucci E, Salaffi F, Di Geso L, Grassi W (2011). Differential diagnosis between rheumatoid arthritis and psoriatic arthritis: the value of ultrasound findings at metacarpophalangeal joints level. Ann Rheum Dis.

[45] Macía-Villa C, Falcao S, Gutierrez M, Medina J, Hammer HB, De Miguel E (2019). Peritenon extensor tendon inflammation in psoriatic arthritis is an enthesitis-related lesion. J Rheumatol.

[46] McGonagle D, Lories RJ, Tan AL, Benjamin M (2007). The concept of a "synovio-entheseal complex" and its implications for understanding joint inflammation and damage in psoriatic arthritis and beyond. Arthritis Rheum.

[47] François RJ, Braun J, Khan MA (2001). Entheses and enthesitis: a histopathologic review and relevance to spondyloarthritides. Curr Opin Rheumatol.

[48] Terslev L, Naredo E, Iagnocco A (2014). Defining enthesitis in spondyloarthritis by ultrasound: results of a Delphi process and of a reliability reading exercise. Arthr Care Res (Hoboken).

[49] Maksymowych WP, Mallon C, Morrow S (2009). Development and validation of the Spondyloarthritis Research Consortium of Canada (SPARCC) Enthesitis Index. Ann Rheum Dis.

[50] Healy PJ, Helliwell PS (2008). Measuring clinical enthesitis in psoriatic arthritis: assessment of existing measures and development of an instrument specific to psoriatic arthritis. Arthritis Rheum.

[51] Gisondi P, Tinazzi I, El-Dalati G (2008). Lower limb enthesopathy in patients with psoriasis without clinical signs of arthropathy: a hospital-based case-control study. Ann Rheum Dis.

[52] Gutierrez M, Filippucci E, De Angellis R (2010). Subclinical entheseal involvement in patients with psoriasis: an ultrasound study. Semin Arthr Rheum.

[53] Kucybała I, Urbanik A, Wojciechowski W (2018). Radiologic approach to axial spondyloarthritis: where are we now and where are we heading?. Rheumatol Int.

[54] Dallaudière B, Dautry R, Preux PM (2014). Comparison of apparent diffusion coefficient in spondylarthritis axial active inflammatory lesions and type 1 Modic changes. Eur J Radiol.

[55] Tsoi C, Griffith JF, Lee RKL, Wong PCH, Tam LS (2019). Imaging of sacroiliitis: current status, limitations and pitfalls. Quant Imaging Med Surg.

[56] de Hooge M, van den Berg R, Navarro-Compán V (2013). Magnetic resonance imaging of the sacroiliac joints in the early detection of spondyloarthritis: no added value of gadolinium compared with short tau inversion recovery sequence. Rheumatology (Oxford).

[57] Offidani A, Cellini A, Valeri G, Giovagnoni A (1998). Subclinical joint involvement in psoriasis: magnetic resonance imaging and X-ray findings. Acta Derm Venereol.

[58] Erdem CZ, Tekin NS, Sarikaya S, Erdem LO, Gulec S (2008). MR imaging features of foot involvement in patients with psoriasis. Eur J Radiol.

[59] Feydy A, Lavie-Brion MC, Gossec L (2012). Comparative study of MRI and power Doppler ultrasonography of the heel in patients with spondyloarthritis with and without heel pain and in controls. Ann Rheum Dis.

[60] Poggenborg RP, Pedersen SJ, Eshed I (2015). Head-to-toe whole-body MRI in psoriatic arthritis, axial spondyloarthritis and healthy subjects: first steps towards global inflammation and damage scores of peripheral and axial joints. Rheumatology (Oxford).

[61] McGonagle D, Tan AL (2015). The enthesis in psoriatic arthritis. Clin Exp Rheumatol.

[62] Hermann KG, Althoff CE, Schneider U (2005). Spinal changes in patients with spondyloarthritis: comparison of MR imaging and radiographic appearances. Radiographics.

[63] Bennett AN, Rehman A, Hensor EM, Marzo-Ortega H, Emery P, McGonagle DG (2010). The fatty Romanus lesion: a non-inflammatory spinal MRI lesion specific for axial-spondyloarthropathy. Ann Rheum Dis.

[64] Maksymowych WP, Chiowchanwisawakit P, Clare T, Pedersen SJ, Ostergaard M, Lambert RG (2009). Inflammatory lesions of the spine on magnetic resonance imaging predict the development of new syndesmophytes in ankylosing spondylitis: evidence of a relationship between inflammation and new bone formation. Arthritis Rheum.

[65] McQueen F, Lassere M, Østergaard M (2008). Magnetic resonance imaging in psoriatic arthritis: a review of the literature. Arthr Res Ther.

[66] Queiro R, Tejón P, Alonso S, Alperi M, Ballina J (2013). Erosive discovertebral lesion (Andersson lesion) as the first sign of disease in axial psoriatic arthritis. Scand J Rheumatol.

[67] Williamson L, Dockerty JL, Dalbeth N, McNally E, Ostlere S, Wordsworth BP (2004). Clinical assessment of sacroiliitis and HLA-B27 are poor predictors of sacroiliitis diagnosed by magnetic resonance imaging in psoriatic arthritis. Rheumatology (Oxford).

[68] Guglielmi G, Scalzo G, Cascavilla A, Salaffi F, Grassi W (2008). Imaging of the seronegative anterior chest wall (ACW) syndromes. Clin Rheumatol.

[69] Frediani B, Allegri A, Falsetti P (2001). Bone mineral density in patients with psoriatic arthritis. J Rheumatol.

[70] Poggenborg RP, Østergaard M, Terslev L (2015). Imaging in psoriatic arthritis. Rheum Dis Clin North Am.

[71] Puhakka KB, Jurik AG, Egund N (2003). Imaging of sacroiliitis in early seronegative spondylarthropathy. Assessment of abnormalities by MR in comparison with radiography and CT. Acta Radiol.

[72] Heuft-Dorenbosch L, Landewe R, Weijers R (2006). Combining information obtained from magnetic resonance imaging and conventional radiographs to detect sacroiliitis in patients with recent onset inflammatory back pain. Ann Rheum Dis.

[73] Jacobson JA, Girish G, Jiang Y, Resnick D (2008). Radiographic evaluation of arthritis: inflammatory conditions. Radiology.

[74] Jacobson JA, Girish G, Jiang Y, Sabb B (2008). Radiographic evaluation of arthritis: degenerative joint diseases. Radiology.

[75] Canella C, Schau B, Ribeiro E, Sbaffi B, Marchiori E (2013). MRI in seronegative spondyloarthritis: imaging features and differential diagnosis in the spine and sacroiliac joints. AJR Am J Roentgenol.

[76] Klecker RJ, Weissman BN (2003). Imaging features of psoriatic arthritis and Reiter's syndrome. Semin Musculoskelet Radiol.

[77] Vinson EN, Major NM (2003). MR imaging of ankylosing spondylitis. Semin Musculoskelet Radiol.

[78] Taljanovic MS, Hunter TB, Wisneski RJ (2009). Imaging characteristics of diffuse idiopathic skeletal hyperostosis with an emphasis on acute spinal fractures. AJR Am J Roentgenol.

[79] Haroon M, Gallagher P, Fitzgerald O (2015). Diagnostic delay of more than 6 months contributes to poor radiographic and functional outcome in psoriatic arthritis. Ann Rheum Dis.

[80] McHugh NJ (2015). Early psoriatic arthritis. Rheum Dis Clin N Am.

[81] Coates LC (2015). Effect of tight control of inflammation in early psoriatic arthritis (TICOPA): a UK multicentre, open-label, randomised controlled trial. Lancet.

[82] Gladman DD, Mease PJ, Choy EH, Ritchlin CT, Perdok RJ, Sasso EH (2010). Risk factors for radiographic progression in psoriatic arthritis: subanalysis of the randomized controlled trial ADEPT. Arthr Res Ther.

[83] Kane D, Stafford L, Bresnihan B, FitzGerald O (2003). A prospective, clinical and radiological study of early psoriatic arthritis: an early synovitis clinic experience. Rheumatology.

[84] Caso F, Costa L, Atteno M (2013). Simple clinical indicators for early psoriatic arthritis detection. Springerplus.

[85] Tillett W, McHugh N (2012). Treatment algorithms for early psoriatic arthritis: do they depend on disease phenotype?. Curr Rheumatol Rep.

